# Sex-Dependent Effects of Environmental Enrichment on Spatial Memory and Brain-Derived Neurotrophic Factor (BDNF) Signaling in a Developmental “Two-Hit” Mouse Model Combining BDNF Haploinsufficiency and Chronic Glucocorticoid Stimulation

**DOI:** 10.3389/fnbeh.2018.00227

**Published:** 2018-10-09

**Authors:** Adrienne M. Grech, Udani Ratnayake, Anthony J. Hannan, Maarten van den Buuse, Rachel A. Hill

**Affiliations:** ^1^Department of Psychiatry, School of Clinical Sciences, Monash Medical Centre, Monash University, Clayton, VIC, Australia; ^2^The Florey Institute of Neuroscience and Mental Health, University of Melbourne, Parkville, VIC, Australia; ^3^School of Psychology and Public Health, La Trobe University, Melbourne, VIC, Australia; ^4^Department of Pharmacology, University of Melbourne, Melbourne, VIC, Australia; ^5^The College of Public Health, Medical and Veterinary Sciences, James Cook University, Townsville, QLD, Australia

**Keywords:** brain-derived neurotrophic factor, spatial memory, environmental enrichment, hippocampus, corticosterone, stress, neuroplasticity

## Abstract

Neurodevelopmental disorders are thought to be caused by a combination of adverse genetic and environmental insults. The “two-hit” hypothesis suggests that an early first “hit” primes the developing brain to be vulnerable to a second “hit” during adolescence which triggers behavioral dysfunction. We have previously modeled this scenario in mice and found that the combined effect of a genetic hapolinsuffuciency in the brain-derived neurotrophic factor (BDNF) gene (1st hit) and chronic corticosterone (CORT) treatment during adolescence (2nd hit), caused spatial memory impairments in adulthood. Environmental enrichment (EE) protocols are designed to stimulate experience-dependent plasticity and have shown therapeutic actions. This study investigated whether EE can reverse these spatial memory impairments. Wild-type (WT) and BDNF heterozygous (HET) mice were treated with corticosterone (CORT) in their drinking water (50 mg/L) from weeks 6 to 8 and exposed to EE from 7 to 9 weeks. Enriched housing included open top cages with additional toys, tunnels, housing, and platforms. Y-maze novel preference testing, to assess short-term spatial memory, was performed at 10 weeks of age. At week 16 dorsal hippocampus tissue was obtained for Western blot analysis of expression levels of BDNF, the BDNF receptor TrkB, and NMDA receptor subunits, GluNR1, 2A and 2B. As in our previous studies, spatial memory was impaired in our two-hit (BDNF HET + CORT) mice. Simultaneous EE prevented these impairments. However, EE appeared to worsen spatial memory performance in WT mice, particularly those exposed to CORT. While BDNF levels were lower in BDNF HET mice as expected, there were no further effects of CORT or EE in males but a close to significant female CORT × EE × genotype interaction which qualitatively corresponded with Y-maze performance. However, EE caused both sex- and genotype-specific effects on phosphorylated TrkB residues and GluNR expression within the dorsal hippocampus, with GluNR2B levels in males changing in parallel with spatial memory performance. In conclusion, beneficial effects of EE on spatial memory emerge only following two developmental disruptions. The mechanisms by which EE exerts its effects are likely via regulation of multiple activity-dependent pathways, including TrkB and NMDA receptor signaling.

## Introduction

Cognitive impairment is a common symptom in a range of neurodevelopmental disorders, including schizophrenia, major depressive disorder (MDD) and anxiety. In schizophrenia, cognitive impairment occurs in ~80% of cases and includes deficits in learning and memory, which have carry-on effects to social and intellectual functioning (Heinrichs and Zakzanis, 1998; Lewis, 2012). It has been reported that individuals with MDD and anxiety can have cognitive impairments in multiple domains including memory and attention (Gualtieri and Morgan, 2008; McIntyre et al., 2013).

One theory for the pathophysiology of neurodevelopmental disorders is the two-hit hypothesis. The “two-hit hypothesis” postulates that the combination of genetic predisposition and environmental insults during critical periods of development can culminate in significant behavioral disruption in adulthood (Klug et al., 2012). The “first hit” (genetic factor) during development creates a vulnerable brain, and when coupled with the “second hit” (environmental factor) triggers the onset of the disorder (Bayer et al., 1999).

Brain-derived neurotrophic factor (BDNF) is an essential neurotrophin responsible for a broad range of neuronal functions (Adachi et al., 2014) and is associated with neurodevelopmental disorders. Post-mortem studies have reported reduced levels of BDNF and its cognate receptor, Tropomysosin-related kinase B (TrkB) in the prefrontal cortex (PFC) and hippocampus of individuals with schizophrenia (Thompson Ray et al., 2011; Reinhart et al., 2015), suggesting a role of BDNF-TrkB signaling in the illness. There is support in the literature for altered BDNF in humans with depression (Lee and Kim, 2010; Zaletel et al., 2017) and anxiety (Soliman et al., 2010; Castrén, 2014). Stress has been recognized as a major environmental risk factor in the pathophysiology of schizophrenia models (van Os et al., 2010; Brown, 2011; Magariños et al., 2018), and depression and anxiety (Binder and Nemeroff, 2010; Zaletel et al., 2017). We therefore modeled the “two-hit” hypothesis by combining genetic haploinsufficiency in the BDNF gene (1st hit) with adolescent chronic corticosterone treatment (2nd hit). We previously found that these animals show short-term spatial memory deficits (Klug et al., 2012; Hill et al., 2014).

Prolonged corticosterone (CORT) administration in rodents is a well-established model to mimic the physiological parameters of chronic stress and disrupt the HPA axis (Buret and van den Buuse, 2014; Shahanoor et al., 2017). The hypothalamic pituitary adrenal (HPA) axis is the well-conserved control center for the body's stress response. While its role is to moderate the stress response, it can cause damage through prolonged release of glucocorticoids (GC) (Du and Pang, 2015). In humans this is cortisol and the rodent equivalent is corticosterone (Papadimitriou and Priftis, 2009), and these can act in a negative feedback loop to regulate the HPA axis in their respective mammalian systems (Du and Pang, 2015). Dysregulation of this loop can have a range of negative effects upon behavior and cognition. Indeed, in the hippocampus there is a dense expression of glucocorticoid receptors (GR), and it is thought that the excess activity of GC here could be contributing to the cognitive deficits associated with chronic stress (Mirescu and Gould, 2006; Jayatissa et al., 2008; Rainer et al., 2012; Du and Pang, 2015).

For humans, leading a healthy lifestyle or having a “positive environment,” in both the physical and emotional sense, helps prevent and create resilience to neurodegenerative and mental health issues (Maass et al., 2014; Brown et al., 2017; Lee et al., 2018). A recent, comprehensive review by Arango et al. (2018) outlines that environmental risk factors such as poverty, stressful urban environments and negative social interactions such as bullying and abuse during childhood and adolescence can act synergistically to increase susceptibility to developing a neurodevelopmental disorder (Arango et al., 2018). It goes on to demonstrate that a range of interventions, including age-appropriate stimulation, proper nutrition and exercise can be important buffers against neurodevelopmental disorders. Another recent review by Devoe et al. suggested that cognitive behavioral therapy and family therapy are useful in the long-term reduction of attenuated psychotic symptoms (Devoe et al., 2018). This resilience is thought to be linked to a holistic health approach, which includes a “stimulating environment.” A stimulating environment encapsulates many domains, including social, physical, and cognitive. Research in adulthood has found that focus on social groups and music therapy can prevent and alleviate depressive symptoms (Cruwys et al., 2013) and schizophrenia patient outcomes (Fachner et al., 2013; Geretsegger et al., 2017; Erkkilä et al., 2018). This is consistent with the Arango et al. review that argued appropriate stimulation is necessary for a healthy mind (Arango et al., 2018). Positive environments in preclinical animal model research generally refer to environmental enrichment (EE), an experimental protocol that aims to provide the laboratory animals with a habitat with an enhanced sensory environment, in order to stimulate experience-dependent plasticity (Nithianantharajah and Hannan, 2006; Novkovic et al., 2015). Rodent EE studies vary in their protocols to create an enriched environment, and include larger living areas, giving the animals access to toys or other stimulating materials, living in larger social groups, and exercise (Clemenson et al., 2015). This has been found to have positive effects including improved cognitive functioning (Yuan et al., 2012), delay of disease progression (Garofalo et al., 2015) and recovering of disease symptoms, with learning and memory also modulated by EE (Burrows et al., 2015). However, some studies have shown that EE can also have a stressful and negative impact upon laboratory animals, including increased aggression (McQuaid et al., 2012).

It is well established in the literature that the hippocampus has a central role in cognition, is affected in human neurodevelopmental disorders (Lavenex et al., 2006; Barnea-Goraly et al., 2014; Ledoux et al., 2014; Blair et al., 2017), and in rodent studies has been particularly responsive to EE-induced effects (Teather et al., 2002). This is hypothesized to occur through the BDNF-TrkB signaling pathway (Novkovic et al., 2015). BDNF binding to TrkB induces receptor dimerization and subsequent phosphorylation of tyrosine residues (Minichiello, 2009), the most important being 705, 515, and 816. Y705 has been called the initiator of receptor autophosphorylation (Benmansour et al., 2016) and has an overall role in TrkB activation, with the extent of phosphorylation of this residue correlating with TrkB activity levels (Huang and McNamara, 2010). The tyrosine residue 515 (Y515) is the Shc adapter protein docking site (Ambjørn et al., 2013; Benmansour et al., 2016), which catalyzes multiple signaling cascades including pathways involved in learning and memory (Yang et al., 2011). Y816 is linked to the phospholipase (PLC)γ1 pathway, has a role in synaptic plasticity, cell survival and axon elongation (Ming et al., 1999; Atwal et al., 2000; Minichiello, 2009), and contributes to ERK activation (Ambjørn et al., 2013).

Several studies have demonstrated that EE increases BDNF levels in the hippocampus (Cao et al., 2014; Ramírez-Rodríguez et al., 2014; Novkovic et al., 2015) and, consequently exerts its positive effects upon cognition (Novkovic et al., 2015). Conversely, stress has been shown to negatively impact BDNF-TrkB signaling (Buckley et al., 2007). Chronic treatment with corticosterone (CORT) has been shown to decrease levels of BDNF mRNA and protein, as well as intracellular BDNF content (Nitta et al., 1999). Thus, we hypothesized that EE may recover the spatial memory deficit previously found in our two-hit model via regulation of the BDNF-TrkB signaling pathway. This hypothesis was tested by measuring protein expression of mature BDNF, TrkB, and multiple TrkB phosphorylation sites in the dorsal hippocampus.

Dysfunction of the inhibitory circuits and consequently the tilting of the excitatory/inhibitory balance toward over-excitation, is a major contributor to cognitive deficits present in neurodevelopmental disorders (Daskalakis et al., 2002; Heckers and Konradi, 2014; Fee et al., 2017; Selten et al., 2018). Excitotoxicity is characterized by increased extracellular concentrations of glutamate, which overactivate N-methyl-D-aspartate receptors (NMDAR) and allow an excess of Ca^2+^ influx. This activates a range of enzymatic effects that may cause cell damage or even cell death, resulting in a variety of detrimental neuronal and cognitive consequences. NMDAR are heteromeric tetramers consisting of different combinations of NMDAR subunits; usually including one NMDAR-1 (GluN1) subunit and at least one or more GluN2(A-D) or GluN3(A,B) subunits (Paoletti et al., 2013). NMDAR are located both at the pre- and post-synaptic sites, positioning them to play vital roles in long-term potentiation (LTP) and plasticity (Paoletti et al., 2013). Both of these processes are highly implicated in cognitive processes such as learning and memory (Nithianantharajah and Hannan, 2006; Vierk et al., 2014).

The first aim of this study was to investigate whether EE could reverse the spatial memory deficit in our “two-hit” model of BDNF haploinsufficiency and CORT treatment and how this would compare to EE effects in wildtype (WT) controls. The second aim of this study was to investigate any molecular changes to the BDNF-TrkB signaling pathway and NMDAR system in the dorsal hippocampus, and if these were modulated differentially by EE according to BDNF genotype, sex, and CORT treatment.

## Materials and methods

### Animals

Male and female BDNF heterozygous (HET) mice (Ernfors et al., 1994) and WT littermate controls were obtained from a breeding colony at the Florey Institute, Melbourne, Australia. All mice were on a C57Bl/6 background and breeders were originally obtained from The Jackson Laboratory (USA). 10 pairs of breeders were set up of WT female × HET male. Tail tissue samples were sent to Transnetyx (Cordova, TN, USA) for genotyping. Mice were weaned at 3 weeks and WT and HET mice were housed together. Males and females were housed separately, with an average of 3 mice per cage. Offspring were randomized into 8 experimental groups: (1) Group-housed males, water and standard housed (SH) (WT *n* = 7, HET *n* = 7), (2) Group-housed males, CORT, and SH (WT *n* = 11, HET *n* = 10), (3) Group-housed males, water, and EE (WT *n* = 9, HET *n* = 9), (4) Group-housed males, CORT, and EE (WT *n* = 11, HET *n* = 12), (5) Group-housed females, water, and SH (WT *n* = 15, HET *n* = 11), (6) Group-housed females, CORT, and SH (WT *n* = 8, HET *n* = 10), (7) Group-housed females, water, and EE (WT *n* = 10, HET *n* = 10), and (8) Group-housed females, CORT, and EE (WT *n* = 10, HET *n* = 8). No obvious competition or dominance ranking within the groups was observed. No overt aggressive behavior was observed for the EE groups. Six animals per group were used for molecular analysis. Mice had *ad libitum* access to food and water in a temperature controlled room maintained at ~22°C and on a 12/12 h light/dark cycle. All procedures were performed during the light phase. All procedures performed were done according to guidelines set by the National Health and Medical Research Council of Australia and approved by the Florey Institute for Neuroscience and Mental Health Animal Ethics Committee.

### CORT treatment

**Figure 1:**
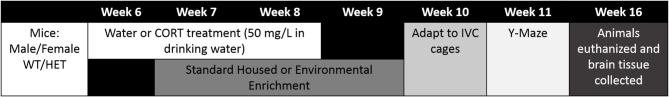
Time line of the experiments. Male and female WT and BDNF heterozygote (HET) mice were used to investigate whether environmental enrichment could ameliorate an established spatial memory deficit in a neurodevelopmental “two hit” model. BDNF heterozygosity was used as the first hit with the second hit being chronic corticosterone (CORT) administered in the drinking water. Environmental enrichment (EE) was administered during and after chronic CORT treatment.

Adolescent/young adult mice were treated with corticosterone in the drinking water from 6 to 8 weeks of age (see Figure 1). These time points were based upon previous studies by our laboratory that show sexual maturation occurs during this period (Hill et al., 2012). Previous research has shown that in mice CORT can be administered between a dose of between 25 and 100 mg/L (Schroeder et al., 2015; Notaras et al., 2017). The CORT concentration of 50 mg/L was chosen with the assumption that the mice would increase water intake as they matured, and this CORT concentration would maintain CORT intake relative to body weight. A high CORT dose has been found in other models to create persistent stress phenotypes, which is important in a chronic model (Johnson et al., 2006; Gourley and Taylor, 2009). Corticosterone hemisuccinate (Q1662-000 Steraloids Inc, United States) was dissolved in water to a final concentration of 50 mg/L. CORT bottles were covered with aluminum foil to be protected from light degradation and were changed every 3–4 days. Bottles were weighed before they were replaced to measure CORT intake by the mice. CORT-treated mice tended to drink between 10 and 20 mg/kg/day. Once treatment stopped at the end of week 8, mice were left undisturbed for another 2 weeks. Control groups received water without CORT.

### Environmental enrichment

Mice received EE from 7 to 9 weeks of age (see Figure 1), during which they were kept in larger open top cages (44 × 30 × 15 cm) with various toys, tunnels, housing and platforms to provide novel cognitive challenges. These were changed once per week. Control mice were housed in open-top standard mouse cages (34 × 16 × 16 cm) with basic nesting materials and were designated “standard-housed” (SH). All mice were given 1 week to acclimatize to their environment when moved from open top to Individually-Ventilated Cages (IVC, 39.1 × 19.9 × 16 cm, Tecniplast, Italy) at the end of week 9.

### Y-Maze short-term spatial memory test

The Y-maze paradigm was performed as previously described (Hill et al., 2014) at week 11. The maze consisted of three arms (30 × 8 × 16 cm) at 120° angles to each other including geometric cues on the far end walls. Briefly, during the initial phase, the mouse was placed into the end of one arm (home arm) and was allowed to explore two arms for 10 min with one arm being closed (novel arm). After a 1 h retention time in the home cage, the mouse was placed into the same Y-maze with all arms open for 5 min. Behavior, including the time spent in each arm, was analyzed with video tracking software (TopScan, CleverSys Inc., Reston, VA, USA). A Discrimination Index (DI) was calculated, which was the amount of time spent in the novel arm divided by the average amount of time spent in the home arm and other familiar arm. Mice with intact spatial memory typically spend more time in the novel arm, reflective of intact memory of the original two familiar arms, and the DI tends to be around 1.5. A DI of around 1.0 represents equal times in all three arms (i.e., chance level) and is interpreted as no recollection of the two arms being familiar.

### Western blot analysis

Mice were killed by cervical dislocation at 16 weeks of age and their brains were collected and stored at −80°C. The hippocampus was bilaterally dissected and separated into dorsal and ventral hippocampus (~50/50). Protein extraction and Western blot analysis were performed as previously described (Hill et al., 2014). Primary antibodies were rabbit anti-BDNF (1:200, Santa Cruz Biotechnology Inc, Santa Cruz, CA, USA or Almone Labs, Israel), rabbit anti-NT-4 (1:200, Santa Cruz), rabbit anti-pTrkB Y705 (1:1,000, Signalway Antibody LLC, Maryland, USA), rabbit anti-pTrkB Y515 (1:1,000, Abcam, Cambridge, MA, USA), rabbit anti-pTrkB Y816 (1:500, Millipore, CA, USA), rabbit anti-TrkB (1:1,000, Santa Cruz), rabbit anti-NMDAR subunit 1 (GluNR1, 1:1,000, Cell Signaling Technology Inc, Danvers, MA, USA), rabbit NMDAR subunit 2A (GluN2A, 1:1,000, Cell Signaling Technology), rabbit NMDAR subunit 2B (GluN2B, 1:1,000, Cell Signaling Technology), or mouse anti-β-actin (1:10,000, Sigma-Aldrich). Secondary antibodies included anti-mouse or anti-rabbit IgG HRP-linked secondary antibodies (1: 2,000; Cell Signaling Technology; Danvers, MA, USA).

### Statistical analysis

All data are expressed as the mean ± the standard error of the mean (SEM). Groups were compared by ANOVA with the independent factors being sex (male or female), genotype (WT or HET), treatment (water or CORT), and environment (SH or EE), using the SYSTAT 13 (Systat Software Inc., San Jose, Ca, USA). Thus, there were 16 experimental groups. *Post-hoc* comparisons were done with Tukey's test. Group differences were considered significant when *P* < 0.05.

## Results

### Y-Maze behavior

**Figure 2:**
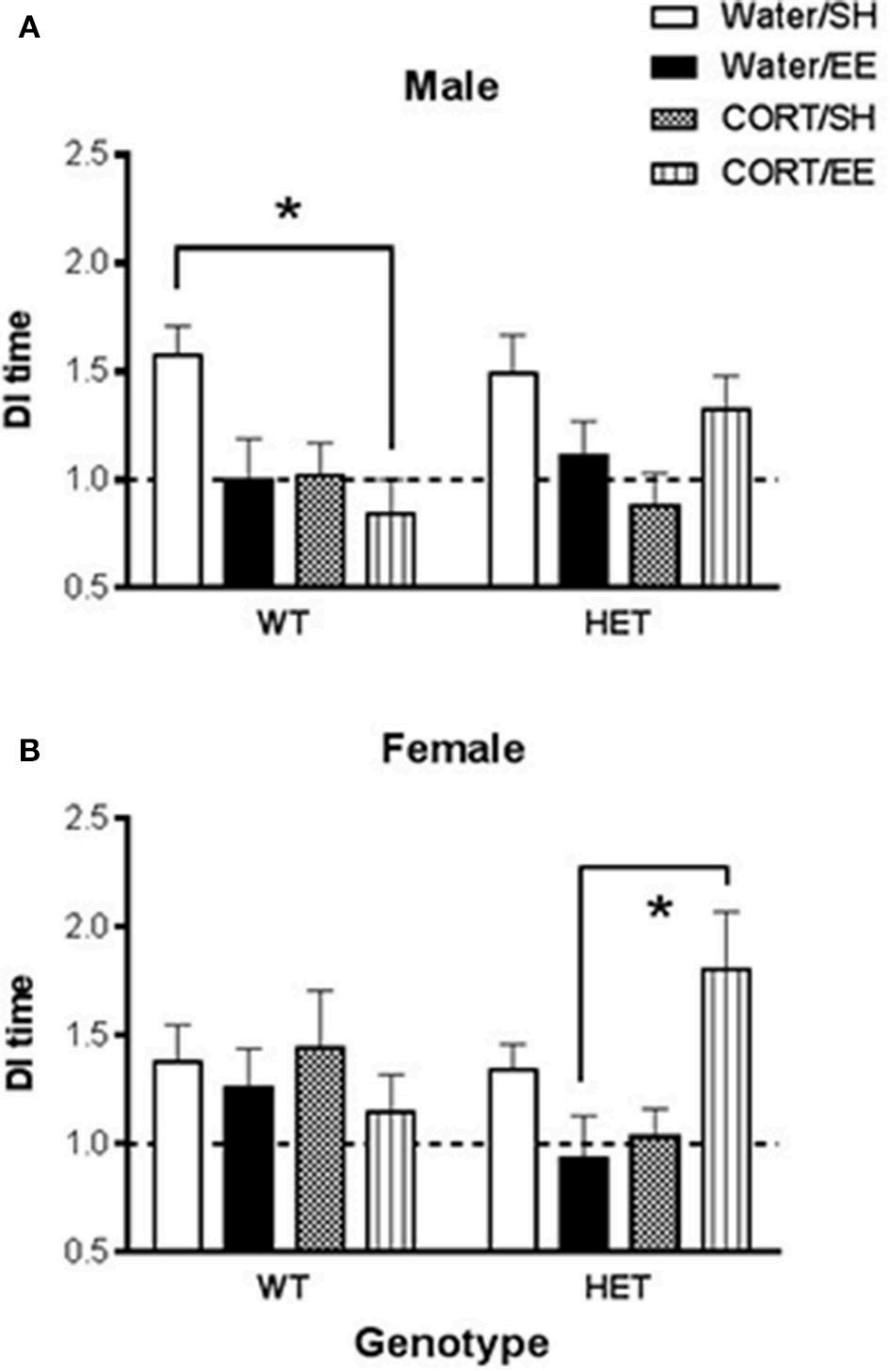
Short-term spatial memory was measured using the Y-maze in wildtype (WT) and BDNF heterozygote (HET) mice which had been treated with chronic corticosterone (CORT), environmental enrichment (EE) or both. **(A)** Shows that male Y-maze behavior was impaired by both EE and CORT with the largest effect seen in male WT mice, but no such additive effect was observed in male BDNF HET mice. **(B)** Shows that female BDNF HET mice exposed to both CORT and EE had significantly higher Y-maze DI than controls. Data are mean ± SEM, *n* = 7–12. *Denotes a significant *post-hoc* interaction of *p* < 0.05.

Univariate ANOVA of the DI of time in the Y-Maze arms revealed that, while there were no main effects of either CORT or EE, there was a significant CORT × EE interaction [*F*_(1, 142)_ = 10.62, *P* = 0.001], suggesting that any effect of CORT depended on whether the animals also underwent EE. Furthermore, this interaction appeared to depend on the genotype of the animals [CORT × EE × Genotype interaction: *F*_(1, 142)_ = 6.81, *P* = 0.010; EE × Genotype interaction, *F*_(1, 42)_ = 5.30, *P* = 0.023]. Because there was also a CORT × Sex interaction [*F*_(1, 142)_ = 5.69, *P* = 0.018], further interrogation of the data was done in males and females separately (Figure 2).

In males, there was again a CORT × EE interaction [F_(1, 68)_ = 7.37, *P* = 0.008] although the CORT × EE × genotype interaction did not reach significance (*P* = 0.079). There was also a main effect of CORT treatment [*F*_(1, 68)_ = 6.31, *P* = 0.014]. Subsequent pair-wise comparison with Tukey's test (Figure 2A) showed that DI values were significantly reduced in WT CORT/EE mice (*p* = 0.045) compared to WT water/SH.

In females, the CORT × EE × Genotype interaction was again significant [*F*_(1, 74)_ = 7.16, *P* = 0.009] suggesting differential effects of EE and CORT depending on the genotype. In HET mice, but not WT mice, pair-wise comparison with Tukey's test revealed that the combination of EE and CORT treatment resulted in significantly higher DI values compared to EE alone (*P* = 0.009) (Figure 2B).

### Molecular results

For the majority of investigated markers there was a main effect of sex when male and female data were combined, so it was decided to analyse the sexes separately. These main effects of sex were: mBDNF: *F*_(1, 67)_ = 30.31, *P* < 0.001; FL-TrkB: *F*_(1, 77)_ = 227.13, *P* < 0.001; Y705 ratio: *F*_(1, 72)_ = 3105.0, *P* < 0.001; Y816 ratio: *F*_(1, 68)_ = 1061.98, *P* < 0.001; GluN2A: *F*_(1, 51)_ = 924.53, *P* < 0.001; and GluN2B: *F*_(1, 67)_ = 9.34, *P* = 0.003.

### mBDNF and NT4

**Figure 3:**
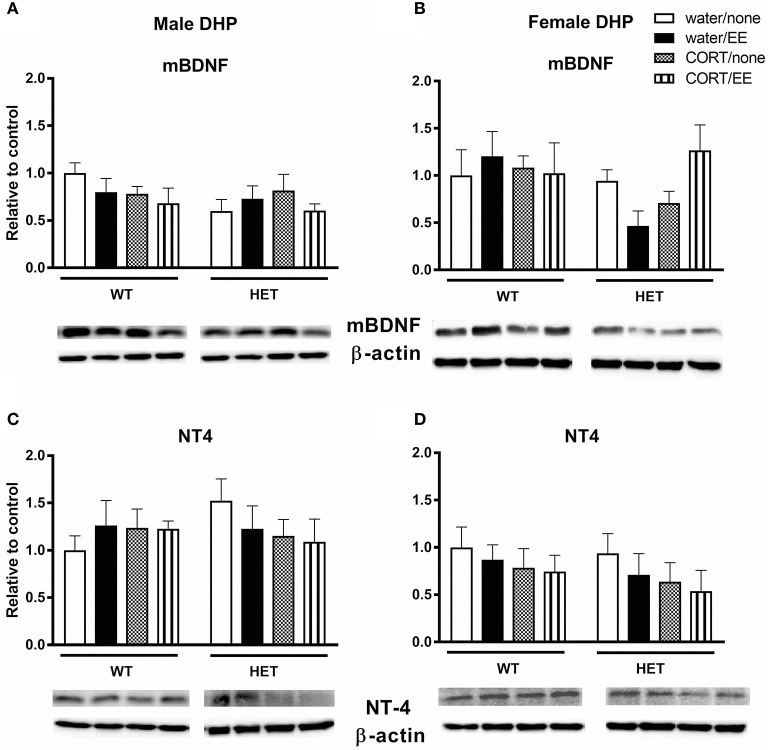
Western blotting was used to measure protein expression of mature BDNF (mBDNF) and neurotrophin 4 (NT4) in the dorsal hippocampus (DHP) of wildtype (WT) and BDNF heterozygous (HET) mice after exposure to chronic corticosterone (CORT), environmental enrichment (EE) or both. Results are presented as standardized protein expression relative to control. There was a close to significant genotype × CORT × EE interaction on mBDNF in female DHP **(B)**. There were no significant changes to protein expression of BDNF in males **(A)** or to NT-4 in males and females **(C, D)**. Data are mean ± SEM, *n* = 3–6, no significant main effects.

Analysis of mBDNF levels revealed no effects of genotype, CORT, or EE on mBDNF expression in male dorsal hippocampus (Figure 3A). In females, there was similarly no main effect of genotype, CORT or EE, however there was a close to significant genotype × CORT × EE interaction [*F*_(1, 27)_ = 4.13, *P* = 0.052]. mBDNF expression levels qualitatively correspond with Y-maze performance with the female BDNF HET + CORT + EE group showing the highest expression (Figure 3B).

Analysis of NT-4 levels showed no effects of genotype, CORT, or EE either in males or females (Figures 3C,D).

### TrkB and pTrkB

**Figure 4:**
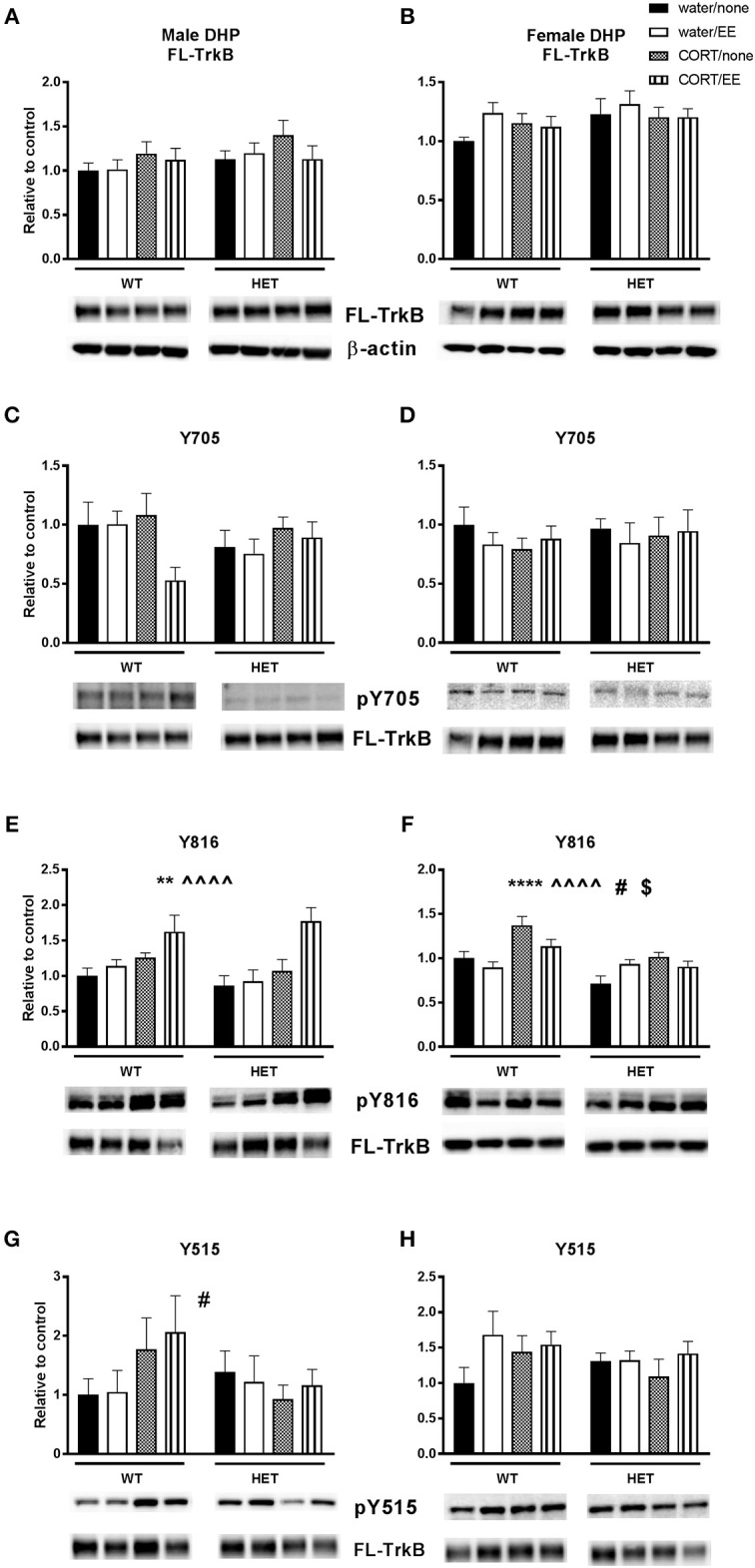
Western blotting was used to measure protein expression of TrkB receptor and phosphorylated TrkB receptor residues after exposure to chronic corticosterone (CORT), environmental enrichment (EE) or both in the dorsal hippocampus of wildtype (WT) and BDNF heterozygous (HET) mice. Results are presented as standardized protein expression relative to control. Phosphorylated TrkB residues are always divided by full length TrkB. For male results refer to **(E)**, which shows a significant effect of CORT (^∧∧∧∧^) and of EE (**) and **(G)** which shows a significant genotype × CORT interaction (^#^). For female results refer to **(F)**, which shows a significant main effect of genotype (**), significant main effect of CORT (^^), significant genotype × EE interaction (^#^) and significant EE × CORT interaction (^*$*^). There were no significant changes to protein expression of FL-TrkB in males and females **(A, B)**, Y705 in males and female **(C, D)** and Y515 in females **(H)**. Data are mean ± SEM, *n* = 4–6, main effect ** *P* < 0.005, ^∧∧∧∧^ or ****P < 0.001, significant interaction ^#^*P* < 0.05 and ^$^*P* < 0.005 for protein expression based on ANOVA. For full details of ANOVA results, see main text.

There were no effects of genotype, CORT or EE or interactions on FL-TrkB in the male and female DHP (Figures 4A,B).

Phosphorylation of TrkB was assessed at positions 705, 816, and 515. There was no main effect of genotype, CORT, or EE or interactions on pTrkB-Y705 in either males or females (Figures 4C,D). With respect to pTrkB-816, in the males there was a significant main effect of CORT [*F*_(1, 36)_ = 16.45, *P* < 0.001] and of EE [*F*_(1, 36)_ = 8.22, *P* = 0.007, Figure 4E], but no main effect of genotype or interactions. Inspection of the data (Figure 4E) shows that both CORT and EE increased the Y816 ratio and appear to have a cumulative effect when co-administered. This cumulative effect was also evident from a close to significant EE × CORT interaction [*F*_(1, 36)_ = 3.82, *P* = 0.058] for Y816.

In female mice, there was a significant main effect of genotype [*F*_(1, 36)_ = 16.87, *P* < 0.001] and CORT [*F*_(1, 36)_ = 18.99, *P* < 0.001], but not EE, on Y816 levels (Figure 4F). There was also a significant EE × CORT interaction [*F*_(1, 36)_ = 5.21, *P* = 0.028], reflecting that CORT tended to increase expression while EE tended to reduce expression of Y816, particularly in WT mice. This differential effect of EE in WT compared to BDNF HET mice was supported by a genotype × EE interaction [*F*_(1, 36)_ = 4.94, *P* = 0.033]. However, there was no genotype × CORT × EE interaction.

Analysis of pTrkB515 expression in male mice showed a significant genotype × CORT interaction [*F*_(1, 39)_ = 4.24, *P* = 0.046, Figure 4G] whereby CORT treatment increased expression in the WT mice, but reduced expression in BDNF HET mice. There was no main effect of genotype, CORT, EE on Y515 in male mice. No significant effects of genotype, CORT, EE or interactions were found in female mice (Figure 4H).

### NMDAR subunits

**Figure 5:**
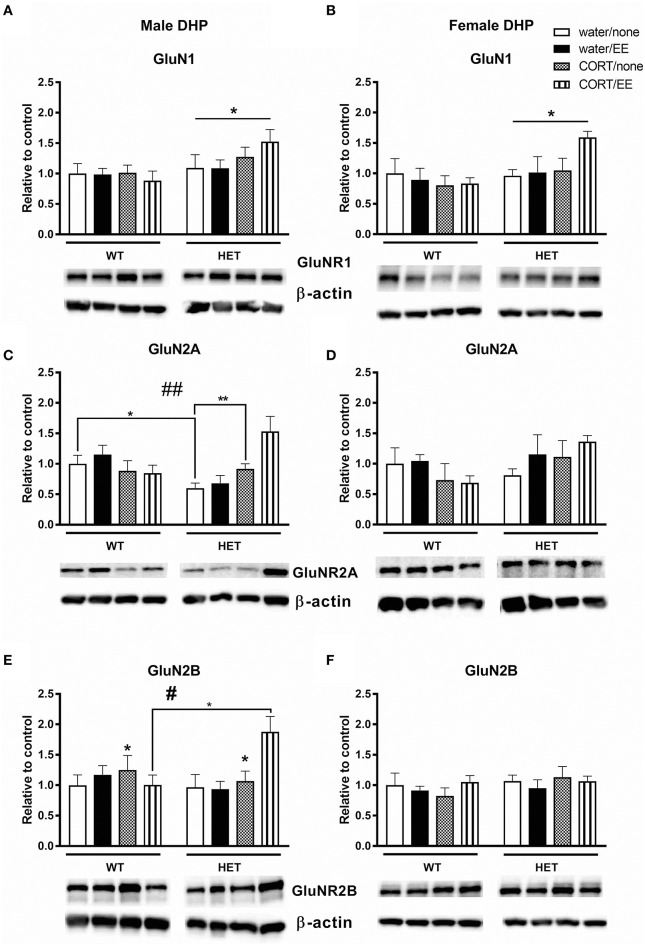
Western blotting was used to measure protein of NMDAR subunits after exposure to chronic corticosterone (CORT), environmental enrichment (EE) or both in the dorsal hippocampus of wildtype (WT) and BDNF heterozygous (HET) mice. Results are presented as standardized protein expression relative to control. **(A,B)** Show protein expression of NMDAR subunit GluNR1, which had a significant main effect of genotype in males and females. **(C)** Shows male GluNR2A protein expression had a genotype × CORT interaction and **(E)** shows male GluNR2B protein expression had significant main effect of CORT and a significant genotype × EE × CORT interaction. There were no significant changes to GluN2A and GlunN2B protein expression in females **(D,F)**. Data are mean ± SEM, *n* = 3–6, main effect or *post hoc* comparison *p < 0.05, significant interaction ^#^*p* < 0.05 and ^*##*^*p* < 0.005 for protein expression based on ANOVA and Tukey's *post-hoc*. For full details of ANOVA results, see main text. **Indicates a significant *post-hoc* interaction of *p* < 0.005.

Analysis of GluN1 protein expression in the male dorsal hippocampus revealed a main effect of genotype [*F*_(1, 39)_ = 5.73, *P* = 0.022] but no effects of CORT or EE or interactions (Figure 5A). Male BDNF HET mice had higher protein expression of GluNR1 compared to male WT mice. Similarly, in females, there was a main effect of genotype [*F*_(1, 28)_ = 4.54, *P* = 0.042], but no effects of CORT or EE, with BDNF HET mice having a higher GluN1 protein expression levels compared to WT mice (Figure 5B).

In male mice, we found a significant genotype × CORT interaction for GluN2A protein expression [*F*_(1, 39)_ = 5.40, *P* = 0.001, Figure 5C) but no effect of genotype, CORT or EE. This significant interaction resulted from reduced GluNR2A protein expression in the male WT CORT/SH group but increased protein expression in male BDNF HET that underwent CORT/SH. *Post-hoc* Tukey's comparisons found while BDNF HET water/SH was significantly decreased (*p* = 0.034), BDNF HET CORT/SH was significantly increased (*p* = 0.003) compared to controls. In female mice, there were no main effects of genotype, CORT, EE or interactions for GluN2A (Figure 5D).

Analysis of GluN2B levels in male dorsal hippocampus revealed a main effect of CORT [*F*_(1, 39)_ = 4.45, *P* = 0.041] whereby CORT increased GluN2B protein expression (Figure 5E). We also found a significant genotype × CORT × EE interaction [*F*_(1, 39)_ = 5.50, *P* = 0.024], and *post-hoc* comparisons found that the BDNF HET CORT/EE group had higher GluN2B protein expression compared to WT CORT/EE (*p* = 0.04). There were no main effects of genotype or EE for GluN2B and no main effects of genotype, CORT, EE or interactions for GluN2B in the females (Figure 5F).

## Discussion

This study investigated the possible preventative benefits of environmental enrichment on memory impairments in a two-hit neurodevelopmental model. The current study is an extension of previous research by our group (Klug et al., 2012), showing in the two-hit model that chronic adolescent stress, modeled here by chronic CORT treatment, combined with BDNF haploinsufficiency leads to a spatial memory deficit in the Y-maze. The primary aim of this study was to investigate if this deficit can be prevented through environmental enrichment. In humans, negative environmental factors can act synergistically with other risk factors to trigger the onset of neurodevelopmental disorders. However, a range of environmental interventions including age-appropriate stimulation, proper nutrition, and exercise have been found to be important preventatives against the onset of neurodevelopmental disorders and mental health issues (Arango et al., 2018).

In the current study, while we anticipated positive modulation on cognition by EE alone, it appeared to have a detrimental effect on spatial memory performance, particularly in male WT mice. In this group, Y-maze DI was reduced in both the EE and CORT groups, with the WT CORT-EE reaching significance compared to the control WT. In contrast, albeit not at the level of a statistical genotype × CORT × EE interaction, in male BDNF HET mice, this additive effect was not observed, and EE/CORT-treated BDNF HET mice had Y-maze DI values not different from control BDNF HET mice. Analysis of data from both sexes combined showed a significant genotype × CORT × EE effect and this interaction remained statistically significant in female mice, where a significantly higher DI was found in the EE/CORT-treated BDNF HET group compared to EE only.

Previous studies examining the effects of EE on hippocampal-dependent memory tasks have shown beneficial effects of EE on long-term memory in the Morris Water maze (Leggio et al., 2005; Garthe et al., 2015) and spatial working memory in the radial arm maze (Leggio et al., 2005). In addition, EE has been shown to improve performance in the novel object recognition task, and this study showed that NR2B transgenic mice with enhanced NR2B function show much longer recognition memory when exposed to EE, and furthermore, they showed increased expression of NR1, NR2B, and NR2A subunits following EE exposure (Tang et al., 2001). This aligns with our study where we found significant effects of EE on NR2B subunits, however, we showed that this effect was specific to males, and these differential effects of EE on NMDA receptor subunits were contingent upon prior exposure to stress and BDNF genotype.

EE exposure for 4 weeks prior to behavioral testing has been shown to enhance spontaneous alternation in the Y-maze paradigm in mice (He et al., 2017) and rats (Jin et al., 2017). In addition, 6 weeks of EE treatment recovered Y-maze spatial memory preference for the novel arm in transient receptor potential channel (TRPC1)–/– mice (Xing et al., 2016). Mice exposed to chronic restraint stress for 4 weeks show impairments in spatial recognition memory in the Y-maze and here, simultaneously living in an enriched environment was able to ameliorate this deficit (Chen et al., 2010).

The above studies consistently show beneficial effects of EE on Y-maze performance, however, we found that EE alone appeared to negatively impact Y-maze novelty preference. This may be due to a number of important methodological considerations. Firstly, it has been shown that including a running wheel in the EE set up is critical particularly to the spatial memory-enhancing effects of EE (Lambert et al., 2005; Rogers et al., 2016). Our protocol did not include a running wheel. In addition, Zeleznikow-Johnston et al. (2017) also used an EE protocol that did not include a running wheel and while they found that EE enhanced visual discrimination and reversal learning, it had no effect on pattern separation or working memory in healthy mice. Thus, it appears that the beneficial effects of EE on hippocampal-dependent spatial memory tasks is heavily reliant upon physical activity as a component of EE. Secondly, the age at which EE is initiated is critical with most previous reports on the beneficial effects of EE initiating EE immediately post-weaning and continuing to maintain EE up to the time of behavioral testing (Simpson and Kelly, 2011). Our study began EE at 7 weeks of age and did not maintain EE until time of testing, thus the mice having experienced an enriched environment would have been in a state of deprivation prior to behavioral testing. Overall, in WT mice EE in our study appears to have functioned as another form of “stress,” with the combined effect of EE and CORT being negatively additive. In contrast, a restorative effect of EE emerged only after CORT treatment in BDNF HET mice. This may represent a variation of the “inoculation hypothesis,” which suggests that EE is a chronic mild stress, and as such creates a resilience to subsequent stressors (Crofton et al., 2015). It should be noted that in our study EE commenced after the introduction of CORT treatment, and mice were returned to standard housing prior to behavioral testing, thus EE followed by standard housing appeared to be another form of stress when compared to standard housing throughout the experimental timeline. Our results would suggest that this mechanism is particularly clear against a background of reduced BDNF levels. Two-hit animals (i.e., BDNF HET mice that received CORT treatment) may have been “inoculated” against EE, and as such EE was restorative to this group.

In the current study we found that Y-maze performance was impaired by chronic CORT treatment in both WT and BDNF HET male but not female mice, suggesting males are more vulnerable to the effects of chronic corticosterone treatment. Female rats and mice are known to have higher levels of circulating corticosterone than males and a previous report in rats found that EE prevented a chronic stress-induced rise in corticosterone in females but not males, and showed a desensitization of the HPA axis to further exposure to an acute stress in female rats (Welberg et al., 2006). This may be due to interactions between female sex hormones such as estradiol and the HPA axis.

In our study, the close to significant genotype × CORT × EE interaction for mBDNF protein expression in the female dorsal hippocampus qualitatively follows the female pattern of Y-maze results, which had a significant genotype × CORT × EE interaction. This was sex-specific, as qualitatively the male Y-maze behavior did not align as closely to the measured mBDNF protein levels. Within the literature there is a broad range of results regarding EE and mBDNF levels. Most likely caused by the wide variety of protocols, studies have shown EE to both increase mBDNF (Cao et al., 2017) and to have no impact (Rogers et al., 2016). The diversity in approach is exemplified with two studies; Cao et al. exposed their mice to EE for 8 weeks, changing the cage every 4 days (Cao et al., 2017), while Rogers et al. (2016) had a similar set up to our study, with 4 weeks of EE and changing the cage weekly. It may be that the frequency of novelty and duration of exposure to EE are major considerations for effects on mBDNF protein expression. The current study found that the water/EE condition did not increase BDNF protein expression, which contrasts with other studies that show EE to increase BDNF (Chourbaji et al., 2012). BDNF is secreted in an activity-dependent manner (Hashimoto et al., 2000). However, in our study at the time-point the brains were collected, the mice were no longer exposed to EE. It could be that EE altered BDNF levels while it was on-going but after EE was stopped BDNF levels returned to pre-EE levels in response to the return to standard housing. In addition, sex is an obvious modifier of BDNF levels and, similar to our findings, a previous report in rats showed that female rats have higher levels of hippocampal BDNF and the effect of EE on increasing hippocampal BDNF levels was greater in females compared to males (Bakos et al., 2009).

In both sexes, both CORT and EE altered the expression of the phosphorylated Y816 TrkB residue. While CORT increased the Y816 ratio in both sexes, EE had a sexually divergent effect whereby it increased the Y816 ratio in males and decreased in females. A recent paper by Bengoetxea et al. (2018) found that only 1 week of EE in male rats increased the expression of TrkB, and improved performance in the Morris Water Maze. Comparatively in our study, when comparing both male genotypes' Y816 ratio to the respective Y-maze data, it would appear that the Y816 ratio shows the inverse trend compared to the Y-maze. This is indicating that while this residue has increased activation, this is not reflective of improved Y-maze performance. However, it should be noted that hippocampal lysates were analyzed 5 weeks following behavioral testing and thus do not reflect TrkB activation at the time of behavioral testing. A similar phenomenon is observed in the females, however it was more genotype specific to the female two-hit mice. Again, while the Y816 ratio is increased, the behavior did not reflect this, perhaps indicating that despite long-term increased activation of the residue this did not result in improved spatial memory performance.

An interesting divergence emerges in the male Y515 ratio results. The recorded genotype × CORT interaction demonstrated that CORT treatment increased Y515 ratio in WT but decreased it in the two hit animals. However, despite this divergence at the molecular level, possibly because of statistical power issues, the analysis did not show a CORT × genotype interaction for spatial memory performance, although this group clearly showed the lowest DI. Because both Y515 and Y816 contribute to the ERK1/2 signaling pathway, it is possible that both are needed to be upregulated to improve behavior.

It has been reported that chronic glucocorticoid treatment disrupts the interaction between glucocorticoid receptors and TrkB with subsequent dampening of the PLCγ signaling pathway (Numakawa et al., 2013), with implications for cognition. Yan et al. (2016) recently demonstrated that chronic CORT treatment decreased pTrkB, and Barfield and Gourley (2017) showed truncated TrkB, the inactive form, to be increased in relation to FL-TrkB after chronic adolescent CORT treatment. Our study adds to this literature by investigating the activation of the distinct residues, and the results discussed above contribute to creating a comprehensive molecular map of how glucocorticoids and the BDNF-TrkB signaling pathway dynamically interact.

Of the three NMDA receptor subunits investigated, the most interesting changes occurred on GluN2B in the males. CORT increased GluN2B protein expression but despite the role of NMDAR in cognition, Y-maze performance was impaired. It could be that this increase of GluN2B is excitotoxic and this is a possible mechanism through which CORT impairs spatial cognition in male mice. This is consistent with previous research by our lab (Klug et al., 2012), which found that CORT treatment increased NR2B protein levels in male BDNF HET mice and paralleled impaired spatial memory as measured by the Y-maze (Klug et al., 2012).

This study was specifically interested in EE as a preventative treatment during adolescence, however a study design limitation is the lack of ability to compare this treatment window with longer-term EE treatment that continues into behavioral testing and up until the point of brain collection. Another limitation of this study is that we present here findings from only one short-term spatial memory task. Future studies should include other spatial memory tasks as well as address other cognitive domains, such as working memory and recognition memory. Overall our study shows that the effects of EE on spatial memory are heavily dependent on BDNF genotype and prior exposure to stress, with our results showing benefit only in two-hit (BDNF HET × CORT) mice. In addition, we show an increased vulnerability of males to chronic CORT and EE and this coincides with male-specific alterations to phosphorylated TrkB residues and NMDA receptor subtypes following both CORT and EE exposure. Female spatial memory performance, however, aligned with mBDNF expression levels in the dorsal hippocampus. These results show that the timing and nature of EE, prior exposure to a model of stress, BDNF genotype, and sex are all critical modifiers of EE-induced spatial memory and molecular outcomes.

## Author contributions

AG performed all Western blot analysis and data analysis and wrote the first draft of the paper. UR performed the behavioral testing and its analysis. AH advised on the original project and on data analysis and interpretation. MvdB designed the project, oversaw behavioral testing, performed data analysis, and edited the manuscript. RH co-designed the project, oversaw Western blot analysis, performed data analysis, and edited the manuscript.

### Conflict of interest statement

The authors declare that the research was conducted in the absence of any commercial or financial relationships that could be construed as a potential conflict of interest.
